# Sensor Based Framework for Secure Multimedia Communication in VANET

**DOI:** 10.3390/s101110146

**Published:** 2010-11-11

**Authors:** Aneel Rahim, Zeeshan Shafi Khan, Fahad T. Bin Muhaya, Muhammad Sher, Tai-Hoon Kim

**Affiliations:** 1 Prince Muqrin Chair for IT Security, King Saud University, Saudi Arabia; E-Mail: fmuhaya@ksu.edu.sa (F.T.B.M.); 2 International Islamic University, Islamabad, Pakistan; E-Mails: zkhan.c@ksu.edu.sa (Z.S.K); m.sher@iiu.edu.pk (M.S.); 3 Center of Excellence in Information Assurance, King Saud University, Saudi Arabia; 4 Management Information Systems Department, College of Business Administration, King Saud University, Saudi Arabia; 5 School of Multimedia, Hannam University, Daejeon, Korea; E-Mail: taihoonn@empas.com (T.-H.K.)

**Keywords:** multimedia, malicious data, security, VANETs, malicious node

## Abstract

Secure multimedia communication enhances the safety of passengers by providing visual pictures of accidents and danger situations. In this paper we proposed a framework for secure multimedia communication in Vehicular Ad-Hoc Networks (VANETs). Our proposed framework is mainly divided into four components: redundant information, priority assignment, malicious data verification and malicious node verification. The proposed scheme jhas been validated with the help of the NS-2 network simulator and the Evalvid tool.

## 1. Introduction

Multimedia communication has attracted the interest of the research community [1]. Multimedia information includes several applications like television, chatting, gaming, internet, video/audio-on-demand, video conferencing, *etc.* [2]. Due to the rapid growth of multimedia applications, security is an important concern [3].

Authentication, confidentiality, integrity and non repudiation are the essential security requirements of multimedia communication in VANETs. [4] Security attacks (denial of service, malicious node attack, impersonation) and vulnerabilities (forgery, violation of copywrite and privacy) exist in multimedia applications due to the mobility and dynamic nature of VANETs [5].

Video transmission in VANETs faces a lot of challenges due to the limited available bandwidth and transmission errors [6]. Security, interference, channel fading, dynamic topology changes and lack of infrastructure are some other factors that degrade the performance of video streaming in VANETs [7].

In this paper we propose a sensor based framework for secure multimedia communication in VANETs. It removes redundant messages and reduces the network load and delays. Malicious nodes and malicious data are easily detected with the help of this framework, which is not possible in existing approaches. It also prioritizes the network and user traffic so high traffic gets more media than lower traffic.

This paper is organized as follows: in Section 2, we will discuss the security issues of multimedia traffic in VANETS and how to detect malicious nodes and data with the help of signal strength and vehicle position. In Section 3, we discuss the proposed framework and the results obtained using the NS-2 simulator is presented in Section 4. Lastly in Section 5 our conclusion is given.

## 2. Related Work

Maxim *et al.* [8] presented the need and importance of security in VANETs. In order to fulfill the security requirements, they proposed a security architecture which will provide security and privacy. VANETs depend on vehicle to vehicle communication, which allows a malicious node to send malicious data over the network. Golle *et al.* [9] proposed a technique to detect and correct the malicious data in VANETs. His technique is based upon the sensor data, collected by vehicles in the VANETs and neighbors information. Redundant information from neighbors and the position of vehicles help detect the malicious data.

Xiao *et al.* [10] proposed a scheme to localize and detect Sybil vehicles in VANETs on the basis of the signal strength. With the help of signal strength a vehicle can verify the position of other vehicles and eliminate the malicious nodes. Xiao first proposed position verification techniques with the help of signal strength but it still has some shortcomings *i.e.*, spoof attacks are possible and data is inconsistent. In order to overcome this weakness, he proposed another solution to prevent malicious nodes in VANETs. Two static algorithms are proposed with the help of traffic patterns and base stations. These algorithms are designed to verify the position of the vehicle and reduce the effect of malicious nodes on communication in VANETs. The following benefits are achieved by using this algorithm:

- Error rate is reduced
- Malicious nodes are easily detected
- It is not hardware dependent

In order to improve performance, selfish or malicious nodes must be captured and removed from VANETs, but it is very difficult to detect these nodes due to the lack of infrastructure and the dynamic nature of VANETs compared to any other *ad-hoc* networks. Raya *et al.* [11] also proposed a feasible framework adapted to the features of the vehicular environment. It detects and prevents the effects of malicious nodes in a VANET scenario.

## 3. Proposed Framework

**Figure 1.**
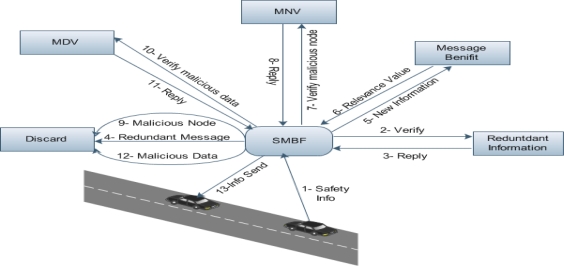
Secure Multimedia Broadcast Framework (SMBF).

Our proposed SMBF framework is composed of four modules: Redundant Information, Message Benefit, Malicious Node Verification (MNV) and Malicious Data Verification (MDV) as shown in Figure 1. SMBF consists of the steps which are given below:

Step 1) Vehicle A wants to share a safety message with Vehicle B
Step 2) SMBF sends message to redundant information for verification
Step 3) On the basis of the reply, SMBF decides to forward or discard the message.
Step 4) Redundant Messages are discarded
Step 5) New Information is sent to Message Benefit
Step 6) Relevance value is sent to SMBF
Step 7) Request to MNV for malicious node verification
Step 8) Receive Reply from MNV and decide to forward or discard the message
Step 9) If the node is malicious, data is discarded
Step 10) Request is sent to MDV to verify the malicious data
Step 11) Receive Reply from MDV and decide to forward or discard the message
Step 12) If the data is malicious, it is discarded
Step 13) If the node and data are not malicious then it is forwarded to Vehicles

**Redundant Information:** Every node maintains a table of Message IDs of currently received messages. We assume that the Message ID is unique and on its basis we detect the redundant messages.

**Message Benefit:** We calculate the priority of each message. Safety Messages get higher priority than any other messages.

**Malicious Node Verification:** We detect the malicious nodes on the basis of signal strength.

**Malicious Data Verification:** We detect the malicious data on the basis of existing messages from neighbors and also on the basis of the position of nodes.

## 4. Implementation and Results

In this study we evaluate the performance of multimedia streaming in a VANET scenario. The mobility model we use is the Manhattan Mobility Model [12] and EvalVid [13] generates the multimedia traffic. We perform the simulation with help of NS-2 [14] on Cygwin [15] and the parameters used in the simulation are listed in Table 1.

### 4.1. Study I

**Figure 2.**
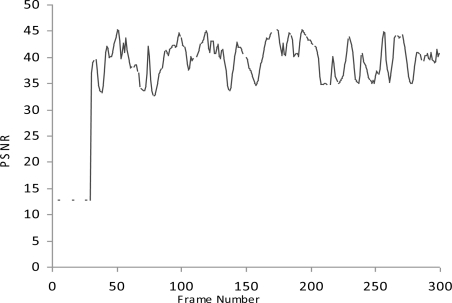
PNSR.

**Figure 3.**
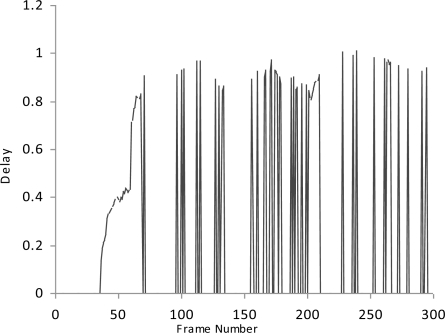
Delay.

**Figure 4.**
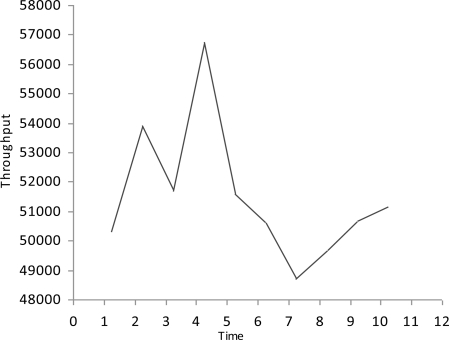
Throughput.

We simulate the multimedia traffic in two different scenarios. First we measure the delay, PSNR and throughput in scenario where there is no mechanism exists for detection of malicious data and malicious node as shown in Figures 2–4.

In this study we have three Vehicles (V1, V2 and V3) that are moving at very high speed.V2 and V3 want to share multimedia traffic with V1 and V2 is a malicious node that sends malicious data to V1 and affects the performance of network. V1 has no framework to determine the validity of data and it considers both V2 and V3 as fair nodes. The delay in this case is higher and throughput is lower because of the effect of malicious data.

### 4.2. Study 2

**Figure 5.**
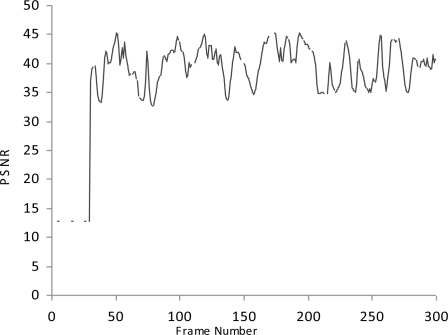
SMBF PSNR.

**Figure 6.**
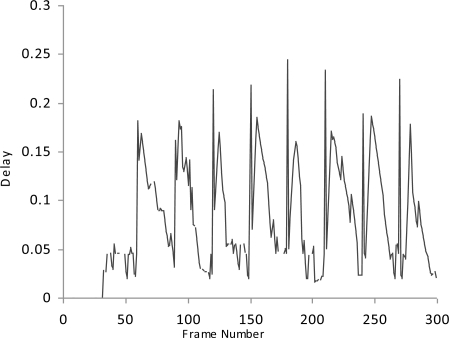
SMBF Delay.

**Figure 7.**
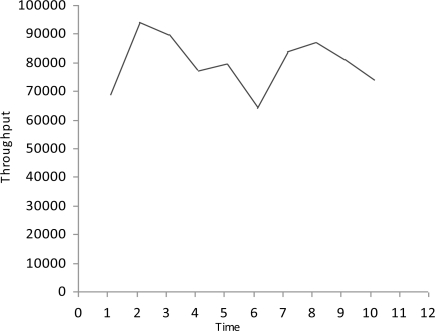
SMBF throughput.

Now we consider the same scenario as the above one. But in this case V1 has the SMBF to determine the redundant messages, malicious nodes and malicious data. We measure the delay, PSNR and throughput by applying the SMBF as shown in Figures 5–7.

Performance of the network is not affected in this case because MDV detects the malicious data on the basis of existing messages from neighbors and also on the basis of the position of nodes, so in this case the delay is lower and throughput is higher because the malicious data does not affect the network.

### 4.3. Comparison

**Figure 8.**
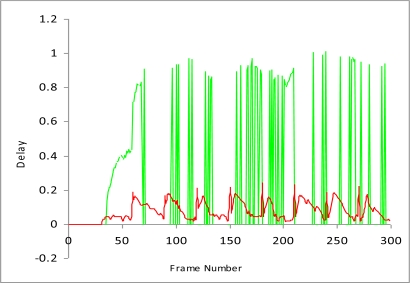
Delay Comparison.

**Figure 9.**
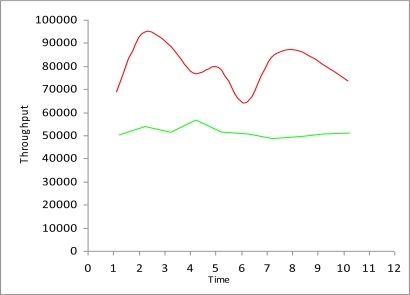
Throughput Comparison.

Now we measure the comparison of study I and study II to determine how much delay increases and throughput decreases, when there is no framework for the detection of malicious data and malicious nodes. Figure 8 and Figure 9 show that delay is much lower when SMBF is applied and throughputs also increase much more when using SMBF. All vehicles have sensors to detect the congestion and improve privacy [16].

## 5. Conclusions

In this paper we have proposed a framework for secure multimedia communication in VANETs. We evaluate the performance of multimedia data in ideal and real scenarios. Simulation shows the performance of multimedia traffic in a VANET scenario. We analyze the affect of malicious nodes and malicious data with and without SMBF. Results show that the performance of multimedia traffic improved while using SMBF.

## Figures and Tables

**Table 1.** Simulation Settings.

**Parameters**	**Values**

Channel	Wireless
Vehicles	3
MAC protocol	802.11
Radio Propagation Model	Two-Ray Ground
Time	50 s
Data type	multimedia
